# RETRACTION: hsa_circ_0077837 Alleviated the Malignancy of Non‐Small Cell Lung Cancer by Regulating the miR‐1178‐3p/APITD1 Axis

**DOI:** 10.1155/jo/9765692

**Published:** 2025-11-06

**Authors:** Journal of Oncology

**RETRACTION**: S. Xu, Y. Jiang, and Y. Duan, “hsa_circ_0077837 Alleviated the Malignancy of Non‐Small Cell Lung Cancer by Regulating the miR‐1178‐3p/APITD1 Axis,” *Journal of Oncology,* no. 2022 (2022), https://doi.org/10.1155/2022/3902832.

The above article, published online on 9 March 2022 in Wiley Online Library (https://www.wileyonlinelibrary.com), has been retracted by John Wiley & Sons Ltd.

The retraction has been agreed following an investigation of the concerns raised by *Actinopolyspora Biskrensis* on PubPeer [1], which identified multiple instances of figure duplication.

Specifically:

Figure 2b: Three panels of the wound healing assay are identical to the panels in Figure 2b of [2], where they depict different cells and experimental conditions.

Figure 2c: The flow cytometry analysis of the Control group shares unexpected similarities with the results shown in Figure 2f (left) of [3], which analyses a different cell type.

Figure 2e: The apoptosis rate of NSCL cells in the over-circ group has unexpected similarities with the flow cytometry analysis of MCF-7 cells with 10 μM of nanoparticles in Figure 2b of [4].

Figure 5h: The plot demonstrating the apoptosis rate of the si-circ-treated NSCL cells is identical to the flow cytometry analysis of the control group in Figure 2b of [4].

As a result of the investigation, the data and conclusions of this article are considered unreliable.

The authors have been informed of this retraction but did not provide a response.

## References

[1] https://pubpeer.com/publications/3D602E73938224D60D36BE76438A93.

[2] Hu Y., Yi B., Chen X., Xu L., Zhou X., Zhu X. (2022). MiR-223 Promotes Tumor Progression via Targeting RhoB in Gastric Cancer. *Journal of Oncology*.

[3] Cheng M., Zhan X., Xu Y. (2022). DNA Methylation of RNA-Binding Protein for Multiple Splicing 2 Functions as Diagnosis Biomarker in Gastric Cancer Pathogenesis and its Potential Clinical Significance. *Bioengineered*.

[4] Fu D., Huang X., Lv Z. (2022). Ultrasound and Magnetic Resonance Imaging of Cyclic Arginine Glycine Aspartic Acid-Gadopentetic Acid-Polylactic Acid in Human Breast Cancer by Targeting αvβ3 in Xenograft-Bearing Nude Mice. *Bioengineered*.

